# Evaluation of Tuberculin Skin Test Positivity and Early Tuberculin Conversion among Medical Intern Trainees in Tunisia

**Published:** 2017

**Authors:** Sonia Toujani, Jouda Cherif, Meriem Mjid, Abir Hedhli, Yassine Ouahchy, Majed Beji

**Affiliations:** El Manar Tunis University, Faculty of Medicine of Tunis, Rabta hospital, Respiratory Department, Research Unit 12SP06, Tunisia.

**Keywords:** Healthcare workers, Medical students, Tuberculin conversion, Tuberculosis

## Abstract

**Background:**

As healthcare workers (HCWs), medical trainees are at a high-risk for acquisition of tuberculosis (TB) infection and disease. To our knowledge, there are no published data about TB infection among medical trainees in Tunisia.

To determine the tuberculin skin test (TST) positivity and tuberculin conversion among a group of medical trainees in different departments at our institution.

**Materials and Methods:**

We performed a prospective study using the TST. The study was conducted in two steps: 1) an initial TST survey and 2) an evaluation of the TST conversion rates.

**Results:**

Among 114 participants, the TST was positive (≥10 mm) in 26.3% and negative (<5 mm) in 57%. The conversion rate of TST was 4%, which was only observed among the trainees assigned to the pulmonary departments. The significant predictor variables of TST positivity were a history of nosocomial TB exposure and training in a high-risk area.

**Conclusion:**

Despite the small number of participants, the high TB conversion rate among the trainees is alarming. This population represents an important target group for a latent tuberculosis infection screening program in countries with limited resources such as Tunisia.

## INTRODUCTION

Active tuberculosis (TB) infection in healthcare settings is recognized as an important occupational risk for HCWs (1–3). The infection control measures recommended in the guidelines of the Centers for Disease Control and Prevention (CDC) are effective in reducing nosocomial *Mycobacterium tuberculosis* transmission (4–6). In high-income countries, these guidelines have been successfully implemented in healthcare settings, but in most low-income countries, these control measures are insufficient. Despite the resurgence of interest in occupational latent tuberculosis infection (LTBI) and TB during the past decade, relatively few recent studies have been published, and most of these studies did not report on the rate of TST conversion. Further, limited data are available regarding LTBI among newly employed HCWs, medical students, and interns (7,8). Moreover, there are currently no proper preventive measures for LTBI (9, 10).

In Tunisia, the incidence of TB has been estimated at 32/100,000 population (11), and testing for LTBI is not mandatory. The National Tuberculosis Control Program in Tunisia recommends preventive treatment only for persons with human immunodeficiency virus (HIV) infection and children aged <5 years who are the household contacts of persons with sputum smear-positive pulmonary TB. There is no recommendation for chemoprophylaxis among HCWs. To our knowledge, there are no published data of LTBI about HCWs and medical students in Tunisia. Screening for LTBI in this population represents an elementary aspect of most hospital infection control programs.

Thus, we performed a prospective study using TST to determine the TST positivity and tuberculin conversion among a group of medical intern trainees in different departments of the medical training hospitals of Tunis.

## MATERIALS AND METHODS

### Population and study design

A prospective survey using demographic and clinical data was performed. After 5 years of medical studies, medical students attend 2 years of an internship, bestowing the status of “internal trainees” or “interns,” and rotate between the various medical, laboratory, and surgical departments for 4 months each. A list of interns attending different wards during the study was provided to us by the director of studies and training. During the study period (July 1–October 31, 2014), 250 interns, who were attending at the three main physician training hospitals of Tunis, participated as volunteers in the study. Only 124 of the trainees had contact with patients, and they had been referred to participate in the study by a pulmonologist.

The internal trainees who had a history of TB, received immunosuppressive therapy, or been absent at the beginning of the survey were not included. The eligible interns were informed about the details of the study and signed a written consent form before participating in the study.

The study was conducted in two steps: in the first step, the TSTs were performed, and in the second step, TST conversions were identified. Each participant answered a standardized questionnaire developed by the study team at the beginning of the internship term. Then, a first TST (TST1) was performed. Students with TST1 <15 mm were retested 4 months later (end of the training rotation) to identify TST conversions. In addition, a new questionnaire was given soliciting information about TB exposure during the 4-month rotation. The participants were given information on the management and screening of positive TB symptoms and the need for further evaluation following a positive TST result.

An induration sized ≥10 mm was considered positive, between 5 mm and 9 mm was intermediate, and <5 mm was negative. TST conversion is presumptive evidence of acquired LTBI and a potential risk for progression to TB disease. We defined conversion as a negative finding on the TST1 with an increase of 10 mm on the second TST (TST2) (12). We considered the pulmonology, emergency, and infectious diseases wards, where possible exposure to *M. tuberculosis* is common, as high-risk areas of TB transmission. Other wards such as surgery, gastroenterology, orthopedics and endocrinology, gynecology, were considered as middle- or low-risk areas. In the pulmonary department, TB patients in the initial phase of treatment, or patients without a confirmed diagnosis were hospitalized. Except for one department, where patients with multi drug resistant TB are hospitalized, no currently proper preventive measures for nosocomial TB infection were in place. Mechanical ventilation and ultraviolet germicidal irradiation (UVGI) were not available in all TB treatment centers. TB and other facility users shared the same waiting area, and there was an insufficient use of protective equipment such as respirators.

### Data collection

Data were obtained through the standardized questionnaire given to the eligible subjects. Data collected included demographic characteristics and clinical information. Prior bacillus Calmette-Guérin BCG vaccination was determined by the presence of a vaccination scar on the right arm.

### Tuberculin skin test administration

TST was administered using the Mantoux method by trained HCWs. A standard dose of tuberculin (*Tuberculin Purified Protein Derivative (PPD), Japan BCG Laboratory*) was injected intradermally into the inner surface of the foramen using a plastic disposable syringe with a short, beveled needle. A tense, pale wheal with a diameter of 6 mm to 10 mm appeared over the needle bevel. When the wheal was less than 6 mm in diameter, the test was administered again. The final step included recording information, reminding the participant about the return visit, and providing education. The transverse induration was measured with a ruler and recorded in millimeters between 48 and 72 hours after injection (12).

### Screening for TB and LTBI

All participants were asked about TB symptoms and TB history.

The following participants were referred for assessment to a pulmonologist:

- Participants with TST conversion and TST1 or TST2 ≥15 mm
- Participants with TB symptoms

Medical assessment included a review of symptoms suggestive of possible active TB, risk factors for TB, and chest X-ray ± bacilloscopy.

Therapy for TB was administrated to participants who developed TB.

Then, if active TB was excluded, and if appropriate, therapy for LTBI, in accordance with current guidelines, was recommended for TST-conversion cases (12).

### Ethics

The study was approved by the local ethics committee of Rabta teaching hospital. Eligible subjects were informed by the investigator about the rationale and aims of the survey. All participants provided written consent, and their personal information was protected.

### Statistical analysis

We used Excel 2007 to enter all the information collected through the questionnaire and the TST results. Then, data were analyzed using STATA software (version 11.1; StataCorp, College Station, USA). The association between the categorical variables and the main outcome of interest, TST positivity, were tested using the Chi-squared test or Fisher exact test. All the categorical variables associated with TST positivity and conversion was added to a multivariate logistic regression analysis to identify independent variables associated with TST positivity and conversion.

## RESULTS

One-hundred fourteen participants underwent TST1 (92% response rate). Ten interns were excluded from the study: five refused to participate, four did not attend their departments, and one had a history of lymph node TB. The mean age of the participants was 27 years (24–30 years), and 66.7% were female. All participants received BCG vaccination at birth. Among the eligible interns, 64% were trained on the pulmonary, emergency, and infectious diseases wards. The main demographic and epidemiological characteristics of the study population are outlined in Table 1.

**Table 1.** The main demographic and epidemiological characteristics of the study population

	**Phase 1 n**	**(%)**	**Phase 2 n**	**(%)**
**Total number of participants**	114	-	100	92
**Mean age, years (SD)**	26.7 (0.1)	-	26.5 (0.1)	-
**Gender**				
Male	38	33.3	32	32
Female	76	66.7	68	68
**BCG immunization**	114	100	100	100
**Tobacco smoking**	8	7	8	8
**Alcohol**	16	14.4	15	15
**Medical history** (asthma, hypothyroidism, rhinitis, anemia, duodenal ulcer)	17	14.9	16	16
**Level for training**				
First year	52	45.6	49	49
Second year	62	54.4	51	51
**Risk areas**				
Low risk areas	41	36	38	38
High risk areas	73	64	62	62
**Previous exposure to active TB**				
NO	67	63.2	63	65
Household	1	1	1	1
Professional	38	35.8	33	34
**Recent professionnal exposure (during the study)**				
**TST result**			61	61
Negative < 5mm	65	57	46	46
Intermediate [5–9mm]	19	16.7	24	24
Positive≥10mm	30	26.3	30	30
**Average TST mm [95%CI]**	5,3 [3,8 – 6.6]		6.1 [4.8–7.3]	

SD: Standard Deviation; TST: Tuberculin Skin Test; 95%CI: 95% Confidence Interval

### Results of TST1

The rate of TST1 positivity was 26.3% and TST1 negativity was 57%; TST1 was ≥15 mm in 10% of samples. The average TST1 induration was 5.3 mm (95% CI: 3.8–6.6). No difference was found between age and sex (p >0.05). The TST results according to the previous training in high-risk areas and clinical level of training are summarized in Table 2.

**Table 2.** TST response stratified by previous training and the level of training

	**TST<5 mm** n=65	**5≤TST<10 mm** n=19	**TST≥ 10 mm** n=30	**p value**	**Conversion** n=4
**Previous training**					
High risk areas n (%)	33 (45.2)	18 (24.7)	22 (30.1)	0.78	4 (100)
Low risk areas n (%)	32 (78)	1 (2.5)	8 (19.5)		0
**Level of training**					
First year n (%)	35 (67.3)	5 (9.6)	12 (23.1)	0.082	2 (50)
Second year n (%)	30 (48.4)	14 (22.6)	18 (29)		2 (50)

TST: Tuberculin Skin Test

### Follow-up and TST conversion

TST2 was performed in 100 trainees. The average of the TST2 was higher than the TST1 (6 mm (95% CI 4.8–7.3; p = <0.001). The highest average was found among trainees in the pulmonary department (7.9 mm (95% CI 6–9.8; p = 0.01). No statistically significant difference was found between age and sex (p >0.05). The association between TST positivity and potential independent variables are shown in Table 3.

**Table 3.** TST positivity and potential independent variables: multivariate logistic regression

**Variables**	**Adjusted OR**	**95% CI**	**p value**
**Gender**	0.57	0.23–1.38	>0 .05
**Age**	0.94	0.64–1.39	>0 .05
**Occupationnel TB contact**	2.55	1.25–6.57	0.04
**Level of training**	1.58	1.13–4.55	0.01
**High risk areas**	2.31	0.61–8.76	0.03

OR : Odd’s Ratio ; 95% CI : 95% Confidence Interval

Between the baseline and follow-up TST, 57% of the participants reported a nosocomial exposure to a potentially infectious patient with *M. tuberculosis*. No participant reported community exposure. TST conversion was documented in four trainees (4%), who had been assigned to a pulmonary department and had reported a recent nosocomial exposure. One of these participants developed pulmonary TB 3 months later. Two participants received therapy for LTBI. According to the multivariate model, TST positivity seemed to be associated with the clinical level of training (p = 0.01; Table 3).

## DISCUSSION

This study was the first one in Tunisia that evaluated TST positivity among interns. The most relevant findings were the high rate of TST positivity at baseline (26.3%) and the relatively high rate of early TST conversion. This result indicates that medical students represent a high-risk population for TB infection. Community rates for a comparable population in Tunisia are unavailable. In high-incidence countries, nosocomial LTBI prevalence ranges from 6.9% to 79% (9,10,13,14). In our study, the TST positivity rate was similar to that found in students in Johannesburg (26.6%) (7). However, the prevalence of TST positive cases among medical students was lower in high-income countries (15–17). The higher prevalence among students could be related to the higher national TB prevalence rate but also to the lack of implemented biosafety measures. In a Brazilian survey, TST conversion rates were twice as high in hospitals without TB infection control measures compared to hospitals with some infection control measures (18). In the present survey, except for one department, there were no currently proper preventive measures for nosocomial TB.

Because all students were vaccinated at birth, the interpretation of the TST results was difficult. Diagnosis of LTBI may be hindered by the non-specificity of TST. BCG vaccination and/or exposure to non-TB mycobacteria, booster phenomenon, and technical issues can contribute to unnecessary chemoprophylaxis (19, 20). Studies in BCG-vaccinated populations have shown a higher prevalence of boosting (8.4%) (21). This booster phenomenon has been associated with BCG vaccination, mostly after infancy and for TST1 reactions of 1–9 mm. One study in Peru suggested that boosting could represent recent LTBI when a 10-mm increment was considered (22). Therefore, true conversion cannot be excluded in this highly exposed population. To avoid a misdiagnosis of TST conversion that is a false-positive due to prior BCG vaccination, a two-step TST in a young BCG-vaccinated populations could be recommended. The PPD response following BCG vaccination varies with the age at vaccination, number of years since the BCG vaccination, number of times vaccinated, and number of PPDs performed. However, an induration of greater than 14 mm is unlikely to be due to prior BCG vaccination. A study from Brazil supports the use of TST as a diagnostic test even in HCWs recently vaccinated with BCG (23).

Use of an interferon gamma release assay (IGRA) has enabled the detection and treatment of LTBI in high group risk. The higher specificity of the Quantiferon TB gold assay (QTB) than that of the TST could prevent unnecessary chemoprophylaxis and detect individuals with true TB infection more accurately (24, 25). However, IGRAs were not available in hospitals where the study was conducted, and these assays are more costly and technically complex than the TST. Replacing the TSTs with the IGRAs as a public health intervention in resource-limited settings is not recommended. Therefore, in our country, the simplicity and low cost of the TST makes it a more feasible screening test. Reviews have suggested that IGRA performance differs in high-TB versus low-TB incidence settings (26). Recently, a large Saudi Arabian study, in a highly diverse HCW population, found that among the TST-positive cases, 54.7% were negative by the QTB (27). Currently, a meta-analysis evaluating the agreement between the TST and QTB in screening for LTBI among HCWs concluded that most of the diagnostic tools had shown low agreement (26). Given the lack of an established gold standard for the diagnosis of LTBI, choosing the proper protocol is a prerogative of the occupational physician. The TST remains the first-step exam, and the QTB is helpful in intermediate- and high-TB burden countries with a high coverage of BCG vaccination (26).

Many risk factors for TST positivity and conversion have been reported such as age, sex, BCG vaccination, and a history of extra-clinical or nosocomial exposure (27–30). In our study, all TST conversion had occurred at the pulmonary wards, and only nosocomial exposure to pulmonary TB patients was identified as a risk factor for TST conversion. Being a contact of an index patient with smear-positive TB was associated with a greater risk of TB infection (31,32). This risk increased with poor ventilation, close contact, and longer duration of exposure. Exposures during cough-inducing procedures were also reported as a risk factor for TB infection (32).

Because TB is considered endemic and many Tunisians are thought to be infected by the time they are young adults, the TST is not currently applied in medical schools in Tunisia. However, we showed that 72.3% of the study participants were not TST-positive at the beginning of the study.

Our results emphasize the necessity of considering TB a priority by health authorities to reduce the risk of nosocomial TB among HCWs. The adoption of individual protection devices, education, and control measures needs to be stressed among medical students. Durando et al. concluded that a follow-up TST is required among students exposed to active TB to reduce nosocomial infection (2).

This study has some limits. The TST data should be interpreted with caution because of the small number of participants and the potential booster effect that may have overestimated the number of converters. Besides, there was a lack of information about the patient contacts of the participants and the use of individual TB protection by the participants.

## CONCLUSION

Measures to control TB infection in healthcare facilities need to be emphasized, and educational programs about TB risks for medical trainees should be implemented in our country. A prospective large-scale study should be performed among Tunisian medical students using the two steps with IGRAs to measure the prevalence of LTBI and to improve the accuracy of these conclusions.
